# Promoting the Emerging Role of Pulse By-Products as Valuable Sources of Functional Compounds and Novel Food Ingredients

**DOI:** 10.3390/foods14030424

**Published:** 2025-01-28

**Authors:** Antonella Lamonaca, Elisabetta De Angelis, Linda Monaci, Rosa Pilolli

**Affiliations:** 1 Institute of Sciences of Food Production, National Research Council of Italy (CNR-ISPA), 70126 Bari, Italy; antonella.lamonaca@ispa.cnr.it (A.L.); linda.monaci@ispa.cnr.it (L.M.); 2 Department of Soil, Plant and Food Sciences, University Aldo Moro-Bari, 70126 Bari, Italy

**Keywords:** pulses by-products, waste valorization, circular economy, functional compounds, innovative foods

## Abstract

The growth of the human population worldwide has increased food demand, generating the massive production of foods and consequently causing enormous production of waste every year. The indiscriminate exploitation of the already limited natural resources has also generated serious environmental and economic crises. The use, or reuse, of waste or by-products represents a viable solution to constrain the problem by promoting alternative routes of exploitation with multiple food and biotechnological applications. This review focuses on the most recent advances in the valorization of food by-products, with specific reference to legume-derived by-products. The main technological solutions for reintroducing and/or valorizing food waste are reported together with a critical discussion of the main pros and cons of each alternative, supported by practical case studies whenever available. First, the possibility to exploit the by-products as valuable sources of functional compounds is presented by reviewing both conventional and innovative extraction techniques tailored to provide functional extracts with multiple food, pharmaceutical, and biotechnological applications. Second, the possibility to valorize the by-products as novel food ingredients by inclusion in different formulations, either as a whole or as hydrolyzed/fermented derivatives, is also presented and discussed. To the best of our knowledge, several of the technological solutions discussed have found only limited applications for waste or by-products derived from the legume production chain; therefore, great efforts are still required to gain the full advantages of the intrinsic potential of pulse by-products.

## 1. Introduction

The progressive shortage of raw materials for world food production has led, in recent decades, to an environmental, economic, and social imbalance, resulting in the indiscriminate exploitation of natural resources, an increase in the cost of foods, and a rise in the number of people in a serious state of malnutrition [1]. These problems have led the world economy to revisit the production system, which is currently based on a linear economy model. The prolonged use of this model has caused the indiscriminate exploitation of the already limited natural resources, resulting in a significant environmental and economic crisis.

One of the main problems is the management of waste that is transported to sanitary landfills every year, thus reducing the availability of land for agriculture and causing damage to health and the environment [2]. Along food supply chains, it is estimated that 14% of food is lost by passing from post-harvesting to retailing [3]. On the contrary, 17% of food turns into waste during distribution and consumption, either in households or in food service [4]. Food waste is estimated to be responsible for 8% of all greenhouse gas emissions, and its value accounts for USD 940 billion in economic losses [5]. Therefore, the use, or reuse, of waste would be a viable solution to constrain the problem, contributing to the food supply of the growing population [6].

The reuse of waste materials represents the foundation of the new model of economy, where ‘linear’ becomes ‘circular’, replacing the system based on ‘*extraction, production, and consumption*’ with a new system of waste prevention, recovery, and regeneration and a balanced use of raw materials and energy [7]. The transition to a circular economy involves different social, economic, and environmental spheres, generating opportunities for regeneration, renewal, and innovation in the agri-food industries [8]. Currently, waste generated by food industries represents the main problem for the management of natural resources, causing shortages, instability in the environment, and damage to the public health by incorrect disposal [9] based essentially on the use of sanitary landfills or incinerators [10]. Despite the diffusion of ‘green’ strategies for waste disposal, the problem unfortunately persists. An in depth-investigation exploring the influence of the characteristics of food products on the most appropriate circular economy strategies for food waste prevention and valorization along food chain has been recently presented by Viscardi et al., 2024. In addition, the same authors described the integration of circular economy practices within food companies, offering detailed guidelines for food waste prevention and valorization [5].

The promoted perspective of waste recovery has prompted the research field to evaluate the potential of agri-food waste products in terms of their chemical composition, in order to re-introduce them as raw material in various industrial processes, fulfilling the demand to move forward to a circular economic model [11].

In 2015, the issues of poverty protection and preservation of the planet described above led the global community to establish a program aimed at resolving these issues: the *Agenda 2030*. The agenda is based on 17 sustainable development goals (SDGs) that refer to different fields of social and economic development. The goals are broken down into 169 targets that are monitored by means of a list of 244 indicators. Specifically, Target 12.5 seeks to substantially reduce the production of waste through prevention, reduction, recycling, and reuse based on the ‘reduce, reuse, recycle’ principle that inspires a circular economy to address waste [12].

## 2. Pulses By-Products: From Waste to Natural Resources of Functional Compounds

In this global perspective, pulses are attracting worldwide attention due to their multiple benefits on the environment and human health; they also represent an excellent and sometimes underutilized alternative to the consumption of meat and meat products [13]. In recent years, thanks to the emerging dietary shift toward conscious, healthy, and low-meat diets, the national production of chickpeas and lentils has increased. European production of dried pulses is close to 95 million tonnes with wide fluctuations in the annual trend. The ranking of the top producing countries sees Russian federation in first place with 4 million tonnes, followed by the United Kingdom, Poland, France, and Germany [14].

Pulses are an essential part of the diet given their high content of protein (up to 57%), carbohydrates (between 40 and 50%), and fiber and fat (between 8 and 15%) [15]. Among the most globally produced and consumed pulses are beans (*Phaseolus vulgaris* L.), chickpeas (*Cicer arietinum* L.), peas (*Pisum sativum* L.), lentils (*Lens culinaris* Medik.), and pigeon peas (*Cajanus cajan* L. Millsp.).

The positive trend of pulse cultivation and production brings along an over-production of waste during the various stages of seed processing. For example, the company Conserve Italia Scarl. (Italy), one of the EU’s largest legume producers and processors, estimates that the quantity of residues originating from the legume agro-industrial pipeline ranges from 5% to 25% of the crop initially harvested [16].

Pulse seed has three distinctive parts, namely the seed coat (8–16%), embryonic axe (1–3%), and cotyledon (80–90%) [17], and removal of the pulse seed coat (dehulling) is the primary process to produce dehulled splits, ground flours, and other fractionated pulse ingredients like pulse protein and fiber. By-product generated from the dehulling process is a mixture of seed coats, embryonic axes, and broken cotyledons [18] and is generally used as low-value ruminant feed with only very limited use in fiber-rich foods [19]. The overall spread of food products enriched with pulse flour for health reasons has led to an expansion of flour production lines, resulting in more hulls being separated from the seeds before the milling stage. Recently, a growing number of studies have suggested that this underutilized by-product has greater potential as a natural source of nutritious dietary fiber, thus promoting its implementation as a functional food ingredient [20].

Recent investigations on the chemical composition of hulls proved that this waste material is rich in nutritional compounds with high biological activity, such as polyphenols, with probable antioxidant and anti-inflammatory activity [18,21,22]. In addition, pulse hulls are the main source of phytochemicals in whole seeds, which are produced by the secondary metabolism of plants [17,23]. Although a few phytochemicals are historically referred to as antinutritional factors, numerous epidemiological studies demonstrated that specific compounds can provide a beneficial effect on human health upon ingestion. Phytic acid, for example, has historically been classified as an antinutritional factor in legumes because of its ability to chelate metal ions such as iron, zinc, and calcium, thus decreasing the absorption of minerals in humans upon ingestion. However, recent studies on mammalian cells contradict this view, indicating that the presence of phytic acid could have protective effects, such as reducing the risk of iron-mediated colon cancer and lowering serum cholesterol and triglycerides in experimental animals [24]. More discussion about the relevance of antinutritional factors in the valorization of pulse by-products will be provided in the following sections.

As general evidence, all the current knowledge about the physico-chemical and nutritional properties of pulse by-products consistently supports and promotes their emerging role as a valuable source of functional compounds and novel food ingredients.

**Figure 1:**
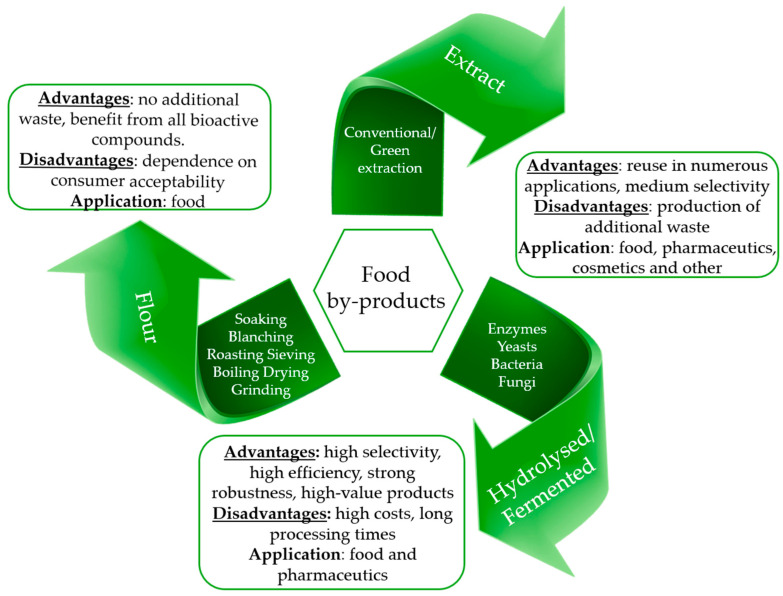
Overview of the different ways to valorize agri-food waste for biotechnological and industrial applications.

There are multiple ways to reuse and recycle the food waste or by-products into potential food, pharmaceutical or biotechnological applications: as source of functional compounds, as (food) ingredients (e.g., inclusion of the whole flour or concentrate), and/or as hydrolyzed/fermented derivates (see Figure 1). Some of these have found so far only a limited application to legume-derived waste or by-products, suggesting great underestimated potentialities that still need to be disclosed and exploited. Each route boasts specific advantages and disadvantages mainly related to the selectivity/applicability of the technological approach and its environmental and economic sustainability (see Figure 1).

The possibility to reuse food (pulse) waste/by-products as sources of functional compounds is strictly related to the intrinsic properties of the matrix under investigation and provides opportunities in terms of development and optimization of extraction techniques (conventional and innovative), as well as potential applications (food, pharmaceutical, biotechnological, etc.) of the derived extracts. The environmental and economic sustainability of this approach, as well as the commercial scalability, should be evaluated case-by-case based on the greenness and applicability of the extraction step, the cost-effectiveness of the protocol (including the recovery step), and the number/amount of further by-products derived from both the exhausted matrix or the extraction step itself.

The reuse of the whole by-products in novel food formulations presents as a main advantage the possibility to realize the full inclusion of the waste/by-product in a new production cycle, benefitting from all the nutritional and functional properties of the matrix itself with no further production of secondary by-products. A potential drawback might rise from the level of consumer acceptability of these innovative formulations, but the renewed awareness and strong sensitivity of new generations toward alternative dietary habits based on conscious, healthy, and sustainable foods pose promising bases for the actual acceptance of novel foods based on valorized by-products [25].

The reuse of food by-products as a substrate for a customized microbiological or enzymatic treatment represents the third main option to valorize food by-products. This route is typically preferred when high selectivity is required to enrich or to increase the availability of a specific class of compounds [26]. Customized fermentation could provide high efficiency, strong robustness, and high-value products with strong application prospects. Moreover, different fermentation types of the same substrate can produce alternative products, targeted by adjusting the operating conditions [27]. In this case, the selection and supply of a proper enzyme or microorganism with consequent optimization of the working conditions represent a big bottleneck, in terms of time and cost-effectiveness of the whole treatment. In addition, the recovery of target products poses important weakness, impairing the sustainability of the entire treatment chain [27].

In the following sections, the three presented approaches are systematically investigated and critically commented on. Specific reference to the reuse of pulse-derived by-products has been included whenever case studies are available. As an alternative, investigations dealing with other fruit and vegetable by-products have been described to support the feasibility of the specific technological approach.

### 2.1. Valorisation of Food By-Products by Selective Extraction of Functional Compounds

Different kinds of agri-food waste obtained from fruits, vegetables, cereal, and legumes can be used as potential source of bioactive compounds, nutraceuticals, and proteins with specific application in various sectors such as pharmaceutical or nutraceutical industries. Bioactive components present in agro-industrial waste can be recovered using various extraction approaches. The plethora of extraction techniques available allows for isolation or enrichment of specific classes of target compounds, with different degrees of purity and selectivity, in various alternative solvents, with strong application prospects.

Currently, extraction technologies have been developed mainly to extract proteins and polyphenol and, to a much lesser extent, other molecules (e.g., fibers or polysaccharides) from agri-food products. These technologies have been demonstrated to also apply to legume by-products and wastes [28,29]. Extraction techniques for food bioactives, including proteins and peptides, are mainly based on conventional solvent extraction techniques and innovative techniques such as supercritical fluid extraction, subcritical water extraction, ultrasound, and microwaves. However, in recent years, a number of alternative non-conventional techniques have been developed for the extraction of biological compounds from food matrices, including Naviglio extraction, high hydrostatic pressure extraction, pulsed electric field extraction, cold plasma extraction, and dry fractionation. A specific review paper summarizing the most recent research on the recovery of phenolic compounds from different food by products by exploiting these emerging techniques has been recently issued [30]. The pros and cons of each technique are detailed along with a critical comparison of their applicability for effective phenolics recovery from food waste. Similarly, the review authored by More et al., 2022, focused on fruit and vegetable waste, deals with the specific application of traditional and green extraction approaches to the recovery of bioactive compounds [31].

In this section, a more general and comprehensive picture of both conventional and non-conventional techniques is provided to assess what has already been applied to the valorization of pulse by-products and what can potentially be explored for this food category.

#### 2.1.1. Conventional Extraction Techniques

The current literature provides already several investigations describing different extraction techniques and their application to the isolation of bioactive compounds from food by-products (see Table 1 for some examples). Most of the conventional techniques depend on the solvent extraction method with potential physical aids such as shaking and/or heating. Various solvents could be used, differing in terms of polarity and pH and depending on the purpose, namely the compounds to be extracted. To increase the extraction yield, a good and common practice is pre-treating the sample before extraction by grinding it to increase the contact surface between sample and solvent. Then, the suitably sized raw material is exposed to different solvents that take up soluble components of interest as well as other flavoring and coloring agents. Samples are usually centrifuged and filtered to remove solid residues, to finally obtain an extract that can be used as it is, namely as an additive or food supplement for the preparation of functional foods or submitted to additional processes to purify or enrich selected compounds.

Although conventional solvent extraction methods are beyond the topic of the most recent review papers due to their low environmental sustainability, many industries still depend on them on account of their economic sustainability. Hereafter, a description of the most common conventional methods for extraction of functional compounds from food and food by-products is provided [32,33].

**Table 1:** Summary of the main application of conventional extraction techniques to the production of functional extracts from food by-products.

Source	By-Product	Technique	Application	Ref.
*Vigna aconitifolia* L.	Generic waste	Soxhlet extraction	Lipid extraction	[28]
Artichoke, lettuce, and cauliflower	Generic waste	Solvent-based extraction	Polyphenols extraction for ready-to-eat soups	[34]
Pomegranate	Peel	Solvent-based extraction	Aqueous extract for shelf life of chicken products	[35]
Grape	Marc	Solvent-based extraction	Cosmetic ingredient	[36]
Citrus	Generic waste	Solvent-based extraction	Cosmetics	[37]
Acerola fruit.	Generic waste	Maceration	Anthocyanin colorant extraction	[38]

The Soxhlet extractor is composed of three fundamental overlapping components: a flask with solvent at the bottom, the extractor with the matrix to be extracted in a thimble made up of filter paper in the middle, and a condenser at the top. The solvent is subjected to boiling, and the vapor passes up through the side tube, is condensed by the condenser, and falls into the thimble containing the matrix. The solvent rich in extracted solutes falls back into the flask to the bottom [32]. Although Soxhlet extraction is a useful technique for many matrices and allows the reuse of the solvent for multiple rounds, it has the disadvantage of heating samples at high temperatures for a long time, destroying thermolabile compounds [39].

Mohammad Hassan Kamani et al. [28] reported an investigation proving the suitability of such an approach to moth bean (*Vigna aconitifolia* L.) milling by-product. Lipids were extracted from the dry flour of bean by-product using a Soxhlet extractor with petroleum ether. The results demonstrated that in terms of lipids, but also proteins and minerals, the by-product had better compositional profiles than whole bean flour.

Maceration is a conventional solvent-based extraction process based on molecular diffusion. The ground matrix is placed in contact with the solvent in a closed vessel at room temperature for sufficient time for the solvent to diffuse through the cell wall to solubilize the solute to be extracted, and the mixture is occasionally agitated to increase diffusion and facilitate extraction [40]. The advantage of this technique is its ease of use (it can be performed by unskilled operators) and the low energy consumption required for the process. On the other hand, different disadvantages, such as the very long extraction times (days or weeks) and the large quantities of solvent consumed, must be considered. Maceration was used to provide information on the structure, molecular weight distribution, and content of tannins in a different set of cowpea seeds selected on the base of seed coat color [41].

Infusion is an extraction similar to maceration. In fact, in both cases, the matrix is left in contact with the solvent in a closed container. However, for the infusion, the solvent is added to the matrix at high temperatures; this makes infusion a faster process than maceration but with higher energy costs [42].

A different technique that allows compound extraction in shorter times without using high temperatures is percolation, although it requires skilled individuals and a large quantity of solvent. In the percolation process, the matrix is moistened with an appropriate amount of solvent and allowed to stand for approximately 4 h in a well closed container. Then, further solvent is added to form a surface layer over the mass, and the mixture is left to macerate in the closed percolator for 24 h. The outlet of the percolator is then opened, and the liquid contained therein is left to drip slowly [43].

Decoction is a very useful technique for extracting non-thermolabile bioactive compounds. In fact, it has the great advantage of using water as an extraction solvent. The process requires the matrix to be placed in water and boiled until the solvent is reduced by 1/4 or 1/8, depending on the molecule to be extracted. Although based on green extraction, decoction is an uncommon technique due to its limited application to thermo-stable compounds [32].

The type of solvent, polarity of functional compounds, and mass transfer rate represent some of the most important crucial factors for the application of these techniques. Although affected by several limitations, as schematized in Table 2, such as toxic solvent intake, high energy requirements, time-consuming processes, adverse effects on the environment with high possibility to degrade the thermally sensitive components, conventional extraction techniques are widely used at the industrial scale due to the low capital investment (instruments and unskilled operators) and easy procedures [44]. In the light of this, a technology readiness level (TRL) with a score of about 9 is often attributed to these extraction approaches [31].

The replacement of the most common toxic solvents with a widespread and consolidated role in conventional extraction techniques with green solvents, such as water and ethanol, has seen only limited application in food by-products valorization [38]. The main constraint for this potential transition lies in the generally lower recovery of functional compounds from food waste provided by green solvent than more toxic alternatives. Indeed, the use of green solvent is typically proposed in combination with emerging technologies where additional chemical-physical aids compensate for the low extraction yield of the solvent itself. Specific comparative investigations were reported in the literature proving this statement. For instance, Avanza et al. evaluated the functional properties of cowpea pods extracts (*Vigna unguiculata* (L.) *Walp*.) using two extraction techniques: pressurized liquid extraction and classic maceration with water. The results demonstrated that the extraction yield with the first method is 23.49% compared with 4.54% obtained with maceration in water. Furthermore, the study showed that the acetylcholinesterase inhibitory activity of the extracts produced with the two techniques is comparable only when using acetone for maceration [45].

However, the feasibility of this technological approach should be considered case-by-case, depending on the specific matrix and technique under investigation, to find some specific applications. For example, the reuse of acerola fruit waste to produce anthocyanin colorants by maceration with water and ethanol has been investigated with promising results. After a proper optimization of the extraction conditions, including an increase in temperature and extraction time, the final functional extract was shown to be satisfactory for the intended use [38].

#### 2.1.2. Alternative Green Extraction Techniques

In recent years, green techniques have gained remarkable attention due to their economic and environmental sustainability and the potential to overcome some of the limitations of conventional approaches. A larger number of experiments have been conducted on the lab-scale using these green extraction techniques, and high value extracts from plant samples have been obtained [46]. Some of the most consolidated green techniques for functional compound extraction are based on supercritical fluid, subcritical water, ultrasound, or microwaves. All of them, have found already promising applications to the valorization of food by-products and specifically to pulse by-products (see Table 3).

Supercritical fluid-based extraction is a selective and sustainable technology that guarantees a high degree of quality and purity of the extracted product. It is commonly used as an alternative to the common extraction with organic solvents to extract, for extracting bioactive compounds from natural sources such as plants, food by-products, and algae [54]. In this technique, a wide range of compounds can be used as solvent, and the selection of the most appropriate supercritical fluids is very important for the correct operation of this process. In the last decade, supercritical carbon dioxide has found a large application in this field. A critical temperature near room temperature (31 °C) and the density of supercritical CO_2_ (at about 200 bar) similar to the density of hexane make this fluid suitable for the extraction of biological materials. In fact, it is possible to extract biological analytes at temperatures around 35 °C as if using a non-polar solvent. The main advantage of this technique is that a small reduction in temperature or pressure causes changes in supercritical conditions and therefore the precipitation of almost all the solute without traces of solvent [55]. In this context, Lima et al. (2019) found that by using super critical CO_2_ modified with ethanol for extraction, higher extraction yields of carotenoids from fruit and vegetable wastes were obtained [47]. Bioactive compounds such as phenols, vitamins, and fatty acids have also been extracted from fruit waste with this green technique [48].

Several trials of industrial scale-up of this technique have already been carried out but only applied to the extraction of functional compounds from foods and plants with good results [56]. In 2009, it was estimated that approximately 300 industrial units used supercritical fluid extraction for obtaining aroma and flavor compounds from solid materials to enrich foods or perfume products reaching a TRL of 9 [31]. However, despite the proved scalability of the supercritical fluid-based extraction, its application to the production of functional extracts from food (pulse) by-waste have been assessed only on the laboratory scale.

Subcritical water-based extraction involves the use of water at temperature and pressure below its critical point (Tc = 374.15 °C, Pc = 22.1 MPa). The characteristic of subcritical water is that its polarity and dielectric constant can be decreased with increasing temperature; therefore, it can behave similarly to methanol or ethanol with proper temperature tuning. This peculiar characteristic allows water to be used as green extraction fluid without any co-solvents, such as acids, alkalis, or organic solvents, for a big variety of organic species [57]. Further, the advantages of this technique are a shorter extraction time, lower solvent cost, and higher quality of the extraction (see Table 4). In addition, subcritical water can significantly improve heat and mass transfer efficiency during extraction, which can increase extraction yield [58].

This technique has been used to extract several bioactive compounds from agri-food waste materials, including legumes (see Table 3). Boroomand (2018) has demonstrated that subcritical water extraction is much more effective than a classic extraction with water for obtaining tannins from faba bean hulls and green pea pods [49].

Ultrasound-assisted extraction is considered a more effective technique than the traditional extraction methods and simpler than supercritical fluids or subcritical water-based alternatives. Ultrasound destroys cell walls, allowing a greater diffusion of the solvent into the cellular materials and consequently improving the mass transfer and the release of analytes from the matrix. The ultrasound frequency and power greatly influence the extraction yield and need to be carefully tuned based on the nature of the material to be extracted [66]. The possibility of using this technology as an “add-on” step to the existing process for extracting molecules from plant and food material has widely increased its application at laboratory levels [50]. Several investigations have been reported that focused on the use of ultrasound-assisted approaches to extract phenolic compounds from cowpea pods [67] and proteins from Ganxet beans [68].

To date, no application of ultrasound-assisted extraction at large-scale industrial has been reported although the eco-extraction platform PEEV (Prestation d’Eco-Extraction et de Vectorisation, France) tried to scale up this technique for industrial extraction process [31]. Anyway, with dedicated investigations addressed to innovate and optimize specific parameters of the process, ultrasound-assisted extraction could have the potential to reach high TRL (9) [31].

Microwave-assisted extraction (MAE) is an extraction technique that uses microwave energy to rapidly heat the solvent/sample mixture to promote the desorption of the analyte, the mass transfer of the analytes from the sample to the solvent, and their solubilization in the solvent. Several comparative studies have demonstrated the excellent performance in terms of recovery and precision of MAE compared to other traditional extraction techniques (for example Soxhlet extraction) and its superiority in terms of reduced solvent consumption (10–30 mL) and extraction times (5–15 min), which put the MAE on the green techniques list [69]. A plethora of natural compounds, such as aromas, antioxidants, colors, phenols, and other primary and secondary metabolites, have been successfully extracted from different matrix and plant-based materials [56]. Focusing on its application to the valorization of agro-industrial waste, MAE was successfully exploited by Cavalluzzi et al. (2022) for the extraction of metabolites from lentil hulls of two selected varieties (Green Eston and Red Crimson). Metabolomic investigations of derived water-based extracts allowed the identification of numerous antioxidant and high nutritional value compounds (flavonoids, peptides, amino acids, and alkaloids) and small bioactive peptides [51]. In addition, in a complementary investigation from the same research group, a higher total phenolic content was observed in ethanolic extracts of lentil hulls obtained with MAE compared to extracts obtained with the traditional maceration technique [52]. It was observed that 7% of all metabolites identified in MAE extracts have proven anti-inflammatory and antispasmodic activities, among which niacinamide, apocynin, and p-coumaric acid were the most abundant [52].

Although several studies performed at the lab or pilot scale (TRL 5 to 7) have demonstrated the potentiality of this technique in the effective extraction of functional compounds from foods, its application at the industrial level is still in the beginning stages. A high TRL (9) could be reached only by investing more efforts in the innovation of the process to meet industrial business needs [31].

Additional approaches based on nonthermal extraction techniques have emerged in recent years thanks to their ecological nature and speed of execution. These include Naviglio extraction, high hydrostatic pressure, pulsed electric field, cold plasma, and dry fractionation (see Table 4 for a detailed comparison of advantages and disadvantages). Naviglio extraction is a solid–liquid extraction based on the new Naviglio principle. Briefly, in the Naviglio extractor, the pressure is increased to 6–8 bar to produce a compression of the solvent on the matrix to be extracted. After a defined time, a steep decompression causes the rapid release of the extraction liquid from the inner part of the solid matrix to the external environment. The solvent mechanically transports the extractable substances contained in the solid matrix to the outside. This technique does not require high temperatures for compound extraction; thus, it is very useful for thermolabile compounds processing [62].

High hydrostatic pressure promotes the release of bioactive compounds from their intracellular compartments, making them more bioaccessible for solubilization. Furthermore, the high pressure increases mass transfer in an immediate response due to the damage caused to the cell membrane, which increases permeability and facilitates secondary metabolite diffusion via solvent extraction [70].

Pulsed electric field extraction is a nonthermal technique that uses a pulse electric field to extract phytoconstituents applied in the food and pharmaceutical industries. This technique has gained more attention in recent years for their potential to extract beneficial materials from food waste/by-products. In addition, pulsed electric field can also be used as a pre-treatment to facilitate the recovery of functional compounds followed by a conventional extraction phase. It is included in the list of green techniques because of the higher yield, reduced solvent and energy consumption, as well as low degradation of phytoconstituents and oils [64]. Interestingly, the application of this technique for bioactive compound extraction from foods is continuously increasing on the industrial scale; indeed, a TRL of 9 is achieved with this technology [31].

Cold plasma extraction is an emerging and relatively unexplored technology with promising applications in various areas of food processing. To date, there are only few investigations on the effect of cold plasma on the production of food- and plant-based functional extracts and their relative biological potential. Most of the reported application focused on the microbial decontamination of food by cold plasma [65].

Finally, the dry extraction technique has found useful applications in the extraction of proteins from foods (legumes and cereals). The main advantages of dry fractionation are the preservation of the native functionality of the proteins and the lower consumption of energy and water (see Table 4 for details). The procedure consists of fine milling of matrix, thus allowing subsequent separation based on the size or density of these particles using air classification and electrostatic separation processes. The fine fraction is rich in proteins, while the coarse fraction is rich in starch [16].

#### 2.1.3. (Bio)technological and Food Applications of Functional Extracts from Food By-Products

The selection of the most sustainable extraction technique for agri-food by-products depends not only on the source and the functional compound to be extracted but also on the potential use of the extract. Operating factors such as extraction yield, temperature, solvent, selectivity, and purity of the final extract strongly affect its final application.

Potentially, the functional extracts resulting from agri-food waste or by-products were already demonstrated to hold strong prospects in multiple fields, such as food, pharmaceutics, or cosmetics.

Extracts rich in bioactive compounds that have been demonstrated to be safe could be used to functionalize foods. For example, aqueous extracts rich in polyphenols derived from artichoke, lettuce, and cauliflower waste were added to ready-to-eat soups up to a concentration of 10 mg/mL. The antioxidant capacity of the soups increased with the addition of the extracts up to 85 times [34]. Unutilized pomegranate peel can be commercialized in the food industry as a potential natural preservative due to its antimicrobial activity. In fact, the addition of aqueous extract of pomegranate peel to chicken products has been demonstrated to improve their shelf life by 2–3 weeks during refrigerated storage [35].

In addition to innovative food formulations endowed with beneficial activity for human health, extracts rich in bioactive compounds can also be used in the pharmacological field. For instance, in 2022, Cavalluzzi et al. demonstrated that the aqueous extract of lentil hulls is a promising source of phytochemicals and nutritional compounds with antioxidant properties. The extract tested on SH-SY5Y neuroblastoma cells provided interesting results for its use as a nutraceutical for the treatment and prevention of neurodegenerative diseases [51]. Moreover, Panaro et al. (2024) verified the anti-inflammatory and antispasmodic properties of lentil hull extract with ethyl acetate using in vitro and ex vivo assays. The extract proved to be useful in the management of inflammatory bowel diseases, usually characterized by inflammation and altered intestinal motility [52].

As for cosmetic field, there are numerous applications using extracts of agri-food by-products to improve the quality of creams, oils, soaps, shampoos, and others. Aqueous olive extracts have been used to develop skin care products due to the proven anti-inflammatory, antioxidant, and detoxifying effects of these extracts on cultured skin cells. A new cosmetic ingredient composed of a fat-soluble fraction of unfermented grape marc has also been formulated to stimulate the production of new collagen and increase the skin’s hydration potential [36]. Finally, citrus by-product extracts, rich in essential oils, are also widely used in cosmetics thanks to their antimicrobial, antioxidant, and anti-inflammatory properties and their ability to modify the melanin content in the skin [37]

Some studies also regarded the design of novel eco-friendly packaging to replace synthetic plastics (edible films, coating, and bio-composites) by recycling the agro-industrial by-products [71]. The fiber residues and antioxidant fraction from artichoke and onion waste were used to produce active and edible alginate-based packaging. The use of eco-friendly packaging led to an improvement in food shelf-life and oxidative stability [53].

### 2.2. Valorisation of Food By-Products by Direct Inclusion as a Food Ingredient

The valorization of agro-industrial by-products as food ingredients encompasses their inclusion as flour and/or a concentrate/isolate obtained by customized treatments, with potential applications in the preparation of various foods, e.g., bread, pasta, cakes, noodles, biscuits, yogurt, cheese, beverages, sports drinks, powdered drinks, fermented dairy products, etc.

The use of by-products as flour has the advantage, compared to extracts, of not producing additional by-products. In addition, the use of the whole by-product provides a large variety of bioactive compounds. By-products can be subjected to different pre-treatments depending on their nature: soaking, blanching, roasting, boiling, drying, grinding, or sieving. Pre-treatment can influence the chemical composition and nutritional and functional properties of the resulting flour.

Dry tissues (dried husk, hull, or seed coat of legumes) are typically ground in millers followed by sieving through sieves [72]. For wet by-products, such as pods of legumes or fruit and vegetable waste, the preparation of flours can start with a blanching or boiling step to inactivate microorganisms and enzymes [73,74]. After blanching, the water is removed by air drying. In 2020, Martínez-Castaño et al. compared the vacuum drying process with air dying process at 60 °C for 8 h for bean pod treatment. The study revealed that characteristics such as color, water-holding capacity, and oil-holding capacity were similar for both methods. Therefore, conventional drying is recommended considering that it is a cheaper and more available process compared to vacuum drying [70].

The pre-treated by-product can then be ground and sieved to produce flour, which is easier to use as a food ingredient. A typical example is provided by the work authored by Canale et al., 2022. In this, case flours of wet artichoke waste (stems and bracts) were used to replace re-milled durum wheat semolina at increasing levels for preparing artichoke waste-enriched bread. Bread with a lower amount of gluten, with a significantly shorter leavening time (thanks to the presence of fermentable dietary fibers such as inulin), and without a significant moisture losses during the five days of storage was obtained [75].

As previously mentioned, by-products need to be pre-treated before use, and the nature of these treatments could affect the functional properties of the resulting ingredient. Functional properties explain how food ingredients behave during preparation and cooking and how they influence the finished food products in terms of consistency, structure, appearance, and flavor [76]. Thus, the evaluation of these properties represents a crucial point as they help to predict the behavior of the ingredient in a specific food system and therefore food quality. The most interesting techno-functional properties to evaluate include solubility, emulsification, foaming, gelling, and the ability to bind water and fat [77].

#### 2.2.1. Direct Inclusion of Legume By-Products: Techno-Functional Properties, Antinutritional Factors, and Consumer Acceptability

Legume by-products due to their versatile techno-functional properties are suitable for many food applications (see Table 5). They have been widely used in baked goods, but their useful techno-functional properties, such as foaming, emulsification, and solubility, make them suitable to produce many other foods including healthy drinks and vegan condiments with a longer shelf-life [72]. The sensory characteristics of the final food obtained from legume waste are also promising for the marketing of these products. The technical-functional properties of foods, such as color and texture, but also other sensory properties, such as flavor and taste, play an important role in determining whether consumers accept the product or not [69]. The color parameters depend on the type and content of natural pigments (e.g., carotenoids, chlorophylls, and phenols) in the final food, as well as on the food processing method. In fact, these compounds are sensitive to heat, air, and pH, which can degrade or transform them [78].

The potential constraints to the consumption of legumes and derived by-product are mainly related to the presence of antinutrients that may limit the availability of various nutrients [82]. Although specific regulations regarding the allowed thresholds of antinutrients in foods are lacking, a general recommendation to keep low consumption levels in the diet still persists, to support the nutritional benefits of legumes [83]. Legume antinutrients include galacto-oligosaccharides, tannins, phytic acid, protease inhibitors, α-amylase inhibitors, saponins, lectins, lathyrogen, cyanogenic glycosides, oxalates, and biogenic amines [84]. The debate concerning the need to remove or retain the antinutritional factors is still open; in fact, many antinutritional factors, which can be harmful at high levels of consumption, might, on the contrary, provide benefits to the human organism, when consumed at low levels. In the previous section, we already discussed the case of phytic acid. Specifically, in addition to its classification as antinutritional compound, it has significant beneficial activities at low concentrations acting as antioxidant, antidiabetic, and antibacterial agent. Moreover, it lowers cholesterol and triglyceride levels in the blood, inhibits the formation of kidney stones, and protects against cancer [85]. Similar findings were reported for alkaloids, which provide positive effects on human health if included in the diet at low concentrations. Cavalluzzi et al. identified low quantities of the alkaloids trigonelline and sinapine [51] in lentil hulls extracts. Trigonelline seems to be involved in heart and liver protection processes and offers benefits in the treatment of hyperglycemia, hypercholesterolemia, nervous and hormonal disorders, and tumors [86], while sinapine is important for its antioxidant and radioprotective activities [87].

In a very recent review paper, Nartea et al. reported that the main pulse antinutrients are located in the cotyledons, with only a small portion of them being present in the seed coats/hulls. Therefore, the content of antinutrients in the dry weight of pulse by-products can be consistently different from the content in the parent edible whole seed [72]. For instance, the phytic acid content in the chickpea seed coat is even 12 times lower than the content in the seed [88], and the trypsin inhibitors responsible for a reduced absorption of dietary proteins are present up to 90% in the cotyledon of faba beans and up to 77% in the cotyledon of chickpeas [89]. Also, α-galacto-oligosaccharide (α-GOS), which causes digestive disorders due to poor absorbance in the small intestine and leading to unpleasant effects such as diarrhea and flatulence [90], is mainly located in the cotyledon [91]. Indeed, despite the common perception, the decorticated legumes do not have increased digestibility compared with whole seeds, since their α-GOS content is not modified by decortication process [91].

Furthermore, most of the common processing methods (soaking, cooking, germination, fermentation, etc.) allow reductions in the final content of antinutritional factors. For example, the content of trypsin inhibitors and tannins (able to bind proteins and amino acids causing their precipitation) can be drastically reduced by thermal treatments, such as boiling of cowpea pods [92]. Extrusion can inactivate trypsin inhibitors without modifying the protein content of fava and kidney beans. [93]. Soaking and germination can reduce the levels of saponins in the seed coatings of black beans [94].

All this experimental evidence could reduce the concerns about the persistence of residual antinutritional factors compared to the benefits of an overall exploitation of pulse by-products in new food formulations. However, to the best of our knowledge, a detailed investigation of the specific antinutritional factors persistent in specific legume by-products is missing.

Several studies have already widely demonstrated that consumer acceptability toward functional foods is influenced by age, cultural environment, social and ethical values, familiarity with the functional ingredient, and above all by taste [95,96,97]. To the best of our knowledge, there is no systematic investigation devoted to trace the consumer perception and acceptability of foods enriched with pulse by-products, but some examples have been reported focused on other agri-food by-products, such as oenological ones [98]. One study reported that some parameters were particularly relevant for the consumer, such as taste, safety, and adherence to traditional products. However, the propensity to purchase bread enriched with wine waste strongly depended on consumer education; specifically, informed consumers are prone to accept enriched foods more easily [98].

Moreover, as general trend, a very recent market analysis focused on the choice of new food products from a circular economy reported that consumers are willing to pay a small premium for products with a hypothetical “circular economy” label, especially if they are educated on sustainability issues and pro-environmental behaviors [25]. Consequently, successful social policies and marketing strategies could be identified and appropriately designed to encourage consumers who are not inclined to purchase functional foods, raise awareness, and inform them on environmental and health issues.

#### 2.2.2. Direct Inclusion of Legume By-Products: Case Studies

The direct inclusion of legume by-products has found already some useful applications in multiple case studies summarized here.

Pea hull was used to enrich Turkish noodles at increasing concentrations. The study showed that the noodles had high dietary fiber content, good amounts of micronutrients, high water absorption capacity, and good swelling volume. Moreover, noodles with pea hulls resulted in a good acceptance by the panelists in terms of sensorial attributes. Therefore, it was concluded that pea hulls have a significant potential as a noodle ingredient [79].

In a study by Costantini et al. (2021), the Italian pasta “orecchietta” enriched with flours of hull derived from Kabuli chickpea or Apulian black chickpeas was characterized by a higher level of bioactive compounds and antioxidant activity compared to the control, thanks to high content of anthocyanins derived from chickpeas hulls. Furthermore, the addition of both Kabuli chickpeas hulls or Apulian black chickpeas hulls positively influenced the color of the final product and improved its firmness and cooking performance [80].

Added-value low-glycemic index noodles were developed using two waste materials: Bengal gram seed coat and broken rice. The noodles produced presented a high content of fiber, both soluble and insoluble, and decreased starch and sugar content compared to the control samples. The glycemic index of the enriched food was significantly lower than the control noodles (56.13 vs. 66.43), suggesting that the inclusion of legumes and cereal by-products could provide valuable help in expanding the range of low-glycemic index foods available to the consumer [81].

Blanched and dehydrated pea pod powder provides a sweet and nutritious ingredient, useful for enriching foods. The powder has in fact been incorporated into egg-free and low-fat mayonnaise as well as instant pea soup powder, widely used by consumers given its ease of use [73,74]. Research shows that the incorporation of pea pod powder leads to an improvement in the structural strength and stability of mayonnaise up to a level of 7.5%, providing acceptable sensory and rheological characteristics [74]. The addition of pod powder to powdered pea soup at a level of 12.5% was found to be acceptable, improving the nutritional quality of the soup without altering its taste and rheological properties. An increase in the content of dietary fiber, protein, and minerals was in fact found, as well as good quantities of carotenoids and chlorophyll [73].

### 2.3. Valorisation of Food By-Products as a Hydrolyzed/Fermented Derivative

Over the years, several strategies have been developed to increase the bioavailability of bioactive compounds in complex food matrices, including physical, chemical, or enzymatic methods. It is known that the compact and insoluble state of many food matrices can constitute a physical barrier to the release and absorption of phytochemicals by the gastrointestinal system, resulting in a limitation to the beneficial effects upon food consumption in the diet [99].

Among others processes, enzymatic hydrolysis represents a promising strategy for selective exploitation of food by-products and their reuse to produce functional foods (see Table 6) [99].

It has been observed that proteolysis is able to influence several properties of food proteins, resulting in an improvement in the beneficial effects due, for example, to a release of polyphenols, increased peptide bioavailability, and reduced content of immunogenic proteins (allergens). Several studies have highlighted the key role played by enzymatic hydrolysis in improving the biological activity of hydrolyzed agro-industrial by-products, such as anti-inflammatory or antioxidant activity [100,101]. Currently, the use of proteolytic enzymes in industrial processes is widespread and represents an advantage compared to chemical processes as it increases the specificity of the hydrolysis and the purity of the product, reducing the environmental impact. Furthermore, it has been observed that food-based hydrolysates obtained from proteolysis induced by certain enzymes under controlled conditions represent an important source of bioactive peptides with numerous biological activities [102].

**Table 6:** Overview of the main applications for food waste recovery by hydrolysis/fermentation processes.

Source	By-Product	Technique	Application	Ref.
Oat	Hull	Hydrolysis with Viscoferm and Ultraflo XL	Hydrolysates with high antioxidant and anti-inflammatory properties	[100]
Wheat	Bran	Hydrolysis with Ultraflo XL	Hydrolysates with improved antioxidant and anti-inflammatory properties	[101]
Lentil	Hull	Hydrolysis with the commercial enzyme preparation Pectinex^®^ Ultra Tropical	Hydrolysates with high antioxidant power	[99]
Tomato	Seed	Fermentation by kefir water culture	Production of bioactive peptides	[103]

In the literature, several studies use the alcalase enzyme to hydrolyze difficult food matrices. The alcalase enzyme is derived from a strain of *Bacillus licheniformis*, and its main enzymatic component is subtilisin A, also known as Subtilisin Carlsberg [104,105]. It is an alkaline serine protease (EC 3.4.21.14) that contains a catalytic triad composed of serine, histidine, and aspartic acid residues in its active site [105]. Regarding the cleavage sites, serine proteases show a preference for aromatic or hydrophobic residues. Due to their ability to break down proteins, alkaline proteases have been widely used to produce protein hydrolysates with high nutritional value [106]. Furthermore, they are added to food and feed mixtures to improve their nutritional value. In medicine, they are used for digestive disorders and food allergies [107].

As reported, lentil protein concentrate treated with the enzyme alcalase induced the hydrolysis of the main protein fractions into smaller peptides within 1 h of treatment at pH 8 and 40 °C [104]. Interestingly, the treatment of the substrate with this enzyme for 1 h at 50 °C at pH 7 produced a reduction of the bands with molecular weights of 47.9, 52.7 and 56.8 kDa, corresponding to the vicilin subunits, and the bands at 22.3 and 39.3 kDa, corresponding to the typical molecular weights of allergenic proteins present in the lentil tegument [108]. The greater hydrolysis capacity of the enzyme alcalase on the lentil matrix has also been confirmed by the studies of Rezvankhah et al. [109] in which a greater degradation of the proteins of green lentils treated with alcalase is reported compared to Flavourzyme. Similar results were also obtained by treating other food substrates such as lupin and pea [104,110].

Fermentation is among the oldest food processes involving enzymes. As known, fermentation is a traditional food processing method widely used to extend the shelf life of foods. Numerous microorganisms are used for the purpose including *Bacillus subtilis*, *Aspergillus oryzae*, etc. [111]. During the fermentation process, microorganisms, whether bacteria, yeasts, or fungi, interact with the food matrix by secreting a large variety of extracellular enzymes, including carbohydrases, lipases, proteases, and others. These molecules promote the splitting of macromolecules into small bioavailable units, leading to significant changes in the physical and chemical properties of the food. On the other hand, cellulases, hemicellulases, pectinases, glucanases, amylases, and esterases are involved in the disintegration of the cell wall, which leads to the release of insoluble bound compounds.

The fermentation technique can be divided into solid-state fermentation and submerged fermentation. In solid-state fermentation, both microbial growth and subsequent product formation occur on a solid substrate in the absence, or near absence, of free water. Filamentous fungi are particularly suitable for this type of fermentation as this technique simulates their natural habitat. At the end of the process, the fermented matrix is submitted to an additional step aimed at extraction of the appropriate compounds.

In recent years, the potential of solid-state fermentation for the treatment of agro-food waste has been re-evaluated by scientists and industries. In fact, this technique offers different advantages, such as easy management of the solid waste, high quality and activity of the extracts, absence of organic solvents, and low downstream refining of the finished product [112]. This technique was found to be advantageous for the synthesis of bioactive compounds and represents an excellent process for industrial use [113]. On the other hand, submerged fermentation involves the cultivation of the microorganism in a liquid medium containing all the nutrients it needs for growth. The synthesized bioactive compounds are secreted into the culture medium by the fermenting organisms during the process. The fermentation of food protein substrates to produce biopeptides using proteolytic microorganisms is an alternative to enzymatic and chemical processes [114]. The submerged fermentation technique has been used for the production of bioactive peptides from various foods, including whey and tomato waste [103,115]. It is important to consider that to produce biopeptides from fermentation, several critical factors must be considered, including optimal operating conditions, substrates, and microorganisms used [116].

Yeasts, fungi, and lactic acid bacteria are the most widely used starter cultures for fermentation. Compared to other microorganisms, lactic acid bacteria have the advantage of being eco-friendly and GRAS (generally recognized as safe), making the fermentation process environmentally friendly and safe [117].

For this reason, there are many applications employing lactic acid bacteria to increase the nutritional value of various food matrices or to isolate biologically active components to be used in functional food formulations.

For example, José Antonio Curiel et al. (2015) evaluated the composition of nineteen traditional Italian legumes fermented with selected lactic acid bacteria (*Lactobacillus plantarum* C48 and *Lactobacillus brevis* AM7) to improve nutritional and functional characteristics. The research showed that the concentrations of free amino acids, soluble fibers, total phenols, gamma-aminobutyric acid (GABA), and antioxidant compounds increased for all legume sourdough starters, while raffinose and tannins decreased [118].

However, other types of bacteria, as well as several yeasts and molds, have recently been used for fermentation processes aimed at increasing digestibility, improving nutritional value, and reducing antinutritional factors in some foods [119,120]. Fungi and yeasts are probably some of the first microorganisms to have been used in fermentation processes aimed at producing compounds of interest for medical, nutritional, and industrial applications [121]. A strain of *Wickerhamomyces anomalus* DSM6766 has already been used in the past to produce sourdoughs for baked products with a low content of 33-mer peptides of celiac disease-related gliadins [122]. Aspartic proteases, which are endopeptidases produced by this yeast, cleave peptide bonds between hydrophobic or aromatic amino acid residues and have maximum activity at acidic pH.

There are several studies in the literature on the use of proteolytic enzymes to improve the technological and nutritional qualities of legumes. In some cases, in fact, it has been shown that treatment with alcalase, endopeptidase, or a mix of exo/endopeptidase improves the antioxidant capacity, solubility, and foaming properties of lentils [123,124,125]. In particular, in 2018, Bautista-Exposito et al. [126] found an increase in the bioavailability of bioactive peptides and phenolic compounds in lentils subjected to treatment with *Lactobacillus plantarum* CECT 748 and the enzymatic preparation Savinase 16 L under alkaline conditions. The hydrolyzed products exhibited improved antioxidant, hypoglycemic, hypolipidemic, anti-inflammatory, and antihypertensive activities compared to the untreated control. Experiments conducted by treating lentils individually with *L. plantarum* CECT 748 and the Savinase preparation have shown that the Savinase enzyme is able to produce mainly bioactive peptides, while *L. plantarum* is responsible for the increase in concentrations of p-hydroxybenzoic acid, vanillic acid, and kaempferol glucosides in the final product, compounds useful for the treatment of metabolic syndrome.

On the contrary, studies on the application of hydrolysis/fermentation of agri-food waste, such as legume hulls, are more limited. Recently, it has been demonstrated that by subjecting red lentil hulls to hydrolysis with the commercial enzyme preparation Pectinex^®^ Ultra Tropical, it was possible to increase the release of oligosaccharides and phenolic compounds from the by-product, demonstrating that the use of hydrolysis processes with selected enzymes increases the antioxidant activity of the hydrolysates despite the insoluble matrix of the hull [99].

## 3. Conclusions

Although all the current knowledge about the physico-chemical and nutritional properties of pulse by-products consistently support and promote their emerging role as a valuable resource, their actual exploitation in a circular economy remains limited. This review presented a comprehensive overview of the different approaches available to reuse legume by-products. Each proposed route and application exhibit a different level of maturity in terms of feasibility, applicability, and scalability on an industrial level. A detailed discussion about the economic and environmental sustainability of the proposed application was not included in this review. However, due to the relevance of these key points for the overall transferability on an industrial scale, a future review would be useful for a complete overview of the topic.

Although considerable progress has been made in recent years in this research area, there are still critical issues that deserve to be investigated. These include the following:

(i) Improve and support the development of green value-added technologies for economic and environmental benefits, in line with sustainable development goals and initiatives. As for agri-food by-products, the application of green extraction technologies, such as supercritical fluid extraction, microwave-assisted extraction, ultrasound-assisted extraction, and others, represents the more promising techniques to be exploited for food waste valorization, due to the numerous advantages and potential sustainability offered by these techniques, such as reduced extraction times, decrease solvent and energy consumption, and improved extraction yields. The use of green solvents, primarily ethanol and water, coupled with sustainable technologies, represents an integrated approach toward the development of extraction processes that could potentially fulfill the recommendations for sustainable extraction procedures. Future challenges are the optimization of parameters, operative conditions, and processes for proper exploitation of these green-technologies to food waste valorization, together with efforts for their scaling-up at the industrial level.

(ii) Deepen the chemical and nutritional characterization of pulses waste-products to evaluate the beneficial potential derived from their reuse counterbalanced by the side-effects of residual antinutritional factors. In this regard, more efforts aimed at investigating and characterizing the nutritional and safety profiles of these by-products in depth are needed. The current literature confirms that antinutrients are mainly located in the cotyledons of legumes, but very few studies provide information about their migration in derived foods when used as high-valuable ingredients. Consequently, it becomes essential to constantly ascertain the actual content of antinutrients in the final food formulation that will be proposed for human consumption. Alternatively, innovative approaches and strategies for eliminating antinutrients from the original pulse by-product or from the final enriched food should be suggested.

(iii) Improve the knowledge of the functional properties of these by-products for their affordable use as ingredients for added value food.

(iv) Consumer acceptability toward the use of new foods enriched with legume waste should also be investigated more systematically to encourage companies to invest in emerging technologies that contribute to the sustainable valorization of these agri-food by-products.

The achievements of these open issues will consolidate the role of pulse by-products as valuable sources of functional compounds and novel food ingredients.

## Figures and Tables

**Table 2:** Comparison of advantages and disadvantages of conventional extraction approaches.

Technique	Advantages	Disadvantages	Ref.
Soxhlet extraction	-Possibility to reuse the solvent	-Uses high temperatures that destroy thermolabile compounds -Long extraction times (hours or days) -Uses large quantities of solvent -Green only if green solvents are used	[32]
Maceration	-Easy to use -Low cost of equipment -Low energy required -Use of low temperatures -Suitable for thermolabile compounds	-Long extraction times (days or weeks) -Uses large quantities of solvent -Low extraction yields -Green only if green solvents are used	[32]
Percolation	-Use of low temperatures -Suitable for thermolabile compounds	-Uses large quantities of solvent -Long times (hours or few days) -Green only if green solvents are used	[32]
Decoction	-Green technique -The solvent is water -Low cost of equipment	-Uses high temperatures that destroy thermolabile compounds	[32]
Infusion	-Faster than maceration -Low cost of equipment	-Uses high temperatures that destroy thermolabile compounds -High energy required -Uses large quantities of solvent -Green only if green solvents are used	[32]

**Table 3:** Summary of the main application of alternative green extraction techniques to the production of functional extracts from food by-products.

Source	By-Products	Technique	Application	Ref.
Fruit and vegetable	Generic waste	Super critical extraction	Carotenoid, vitamin, and phenol extraction	[47,48]
Faba bean and green pea	Hull and pod	Subcritical water extraction	Tannin extraction	[49]
Cowpea	Pod	Ultrasound-assisted extraction	Phenolic compound extraction	[50]
Lentil	Hull	Microwave-assisted extraction	Antioxidant extraction for nutraceuticals	[51]
Lentil	Hull	Microwave-assisted extraction	Anti-inflammatory extraction useful in the management of inflammatory bowel disease (IBD)	[52]
Artichokes and onions	Generic waste	Ultrasound-assisted extraction	Eco-friendly packaging production	[53]

**Table 4:** Comparison of advantages and disadvantages of the alternative green extraction approaches.

Technique	Advantages	Disadvantages	Ref.
Supercritical fluid-based extraction	-Green technique -Use of low temperatures -Suitable for thermolabile compounds -Possibility of using small amounts of solvent -Purity of the extract -Usable on very large or very small quantities of samples -Use of environmentally friendly solvents (CO_2_)	-High cost -Low selectivity for polar compounds if CO_2_ is used -Low extraction yields	[54]
Subcritical water-based extraction	-Green technique -Short extraction time -Easy to optimize by changing only the pressure -Low solvent cost -Use of environmentally friendly solvents (H_2_O) -High quality of the extract -High extraction yield	-Subcritical water management -Cost of the extraction system -Small changes in temperature and pressure greatly modify the selectivity -Intrinsic dilution of the extracts	[59]
Ultrasound-assisted extraction	-Green technique -Simpler than techniques using supercritical fluids or subcritical water -Possibility of combination with other extraction techniques -Ease of execution -Potential for scale-up -Low cost of instrumentation	-Compound degradation due to overheating -Nonselective -Need to optimize different parameters, including time, power, and probe size, to have good yields	[60]
Microwave-assisted extraction	-Green technique -Ease of execution -Very short extraction times (5–15 min) -Use of small quantities of solvent -Low energy consumption -Low cost of instrumentation	-Not suitable for large sample quantities -Nonuniform heating -Phenolic compound degradation due to overheating -Nonselective -Limited scale-up	[61]
Naviglio extraction	-Green technique -Use of low temperatures -Suitable for thermolabile compounds -Short extraction times (about 2 h) -High repeatability	-High costs -Sample dilution after many cycles	[62]
High hydrostatic pressure extraction	-Green technique -Use of low temperatures -Suitable for thermolabile compound -High extraction yield -Purer extract than traditional techniques	-High costs	[63]
Pulsed electric field extraction	-Green technique -Use of low temperatures -Suitable for thermolabile compounds -High extraction yield -Low solvent use -Low energy consumption	-High cost -Extraction dependent on the conductivity of the matrix -Possible transfer of electrode material into the sample -Not suitable for industrial scale -Compound degradation due to overheating	[64]
Cold plasma extraction	-Green technique -High extraction yield -No solvents used	-Few studies on the effect of plasma extraction on the production of food/plant extracts -Small sample quantities -Cost of plasma generation	[65]
Dry extraction technique	-Green technique -Preservation of the native functionality of the proteins -Low solvent use -Low energy consumption	-Only suitable for protein extraction	[16]

**Table 5:** Overview of the main applications for food waste recovery by direct inclusion as a food ingredient.

Source	By-Product	Technique	Application	Ref.
Artichoke	Stem and bract	Direct inclusion as a food ingredient	Bread with low gluten content and a long shelf life	[75]
Pea	Hull	Direct inclusion as a food ingredient	Turkish noodles with high fiber and micronutrients	[79]
Kabuli chickpea and Apulian black chickpeas	Hull	Direct inclusion as a food ingredient	Pasta with high levels of antioxidants	[80]
Bengal gram	Seed coat	Direct inclusion as a food ingredient	Low-glycemic index noodles	[81]
Pea	Pods	Direct inclusion as a food ingredient	Egg-free and low-fat mayonnaise	[74]
Pea	Pods	Direct inclusion as a food ingredient	Instant pea soup powder	[73]

## Data Availability

No new data were created or analyzed in this study.
